# Lung Marginated and Splenic Murine Resident Neutrophils Constitute Pioneers in Tissue-Defense During Systemic *E. coli* Challenge

**DOI:** 10.3389/fimmu.2021.597595

**Published:** 2021-04-19

**Authors:** Goda Juzenaite, Judith Secklehner, Juho Vuononvirta, Yoseph Helbawi, John B. G. Mackey, Charlotte Dean, James A. Harker, Leo M. Carlin, Sara Rankin, Katia De Filippo

**Affiliations:** ^1^ National Heart and Lung Institute (NHLI), Imperial College London, London, United Kingdom; ^2^ Cancer Research UK Beatson Institute, Glasgow, United Kingdom; ^3^ William Harvey Heart Centre, Barts & the London School of Medicine & Dentistry, Queen Mary University of London, London, United Kingdom; ^4^ Asthma UK Centre for Allergic Mechanisms of Asthma, London, United Kingdom; ^5^ Institute of Cancer Sciences, University of Glasgow, Glasgow, United Kingdom

**Keywords:** intravital microscopy, *E. coli* challenge, neutrophil activation, intravascular neutrophils, splenic resident neutrophils

## Abstract

The rapid response of neutrophils throughout the body to a systemic challenge is a critical first step in resolution of bacterial infection such as *Escherichia coli* (*E. coli*). Here we delineated the dynamics of this response, revealing novel insights into the molecular mechanisms using lung and spleen intravital microscopy and 3D *ex vivo* culture of living precision cut splenic slices in combination with fluorescent labelling of endogenous leukocytes. Within seconds after challenge, intravascular marginated neutrophils and lung endothelial cells (ECs) work cooperatively to capture pathogens. Neutrophils retained on lung ECs slow their velocity and aggregate in clusters that enlarge as circulating neutrophils carrying *E. coli* stop within the microvasculature. The absolute number of splenic neutrophils does not change following challenge; however, neutrophils increase their velocity, migrate to the marginal zone (MZ) and form clusters. Irrespective of their location all neutrophils capturing heat-inactivated *E. coli* take on an activated phenotype showing increasing surface CD11b. At a molecular level we show that neutralization of ICAM-1 results in splenic neutrophil redistribution to the MZ under homeostasis. Following challenge, splenic levels of CXCL12 and ICAM-1 are reduced allowing neutrophils to migrate to the MZ in a CD29-integrin dependent manner, where the enlargement of splenic neutrophil clusters is CXCR2-CXCL2 dependent. We show directly molecular mechanisms that allow tissue resident neutrophils to provide the first lines of antimicrobial defense by capturing circulating *E. coli* and forming clusters both in the microvessels of the lung and in the parenchyma of the spleen.

## Introduction

Neutrophils are considered the first line of host defense as they are the most abundant innate immune cell in the blood and among the first to be recruited into tissues during an infection. Parabiosis experiments reveal that even under homeostasis, neutrophils infiltrate almost all tissues (1). Their rate of infiltration and retention varies considerably between tissues. After the bone marrow, the lung and the spleen represent the two major sites of neutrophil retention (1) and evidence suggests that retained neutrophils play an important role in immune surveillance in both of these organs (2).

In the lungs, murine ‘resident’ neutrophils are retained in the microvasculature, where, under steady state, they exhibit a range of migratory behaviors such as crawling, tethering or adherent (3). It has been shown that following systemic infection, neutrophil migration speed increases as they crawl towards and phagocytose *E. coli* initially captured by lung microvascular endothelial cells (4).

While the spleen has traditionally been considered an important site of neutrophil clearance, recent studies have shown that this tissue also has a large population of resident neutrophils in the red pulp, that play a role in the immune response to pathogens. Two sub-populations of neutrophils have been identified under steady state conditions in the mouse spleen (5). Ly6G^high^ neutrophils are mature and motile and Ly6G^int^ neutrophils are immature and more static (5). Interestingly, spleen intravital microscopy (S-IVM) has shown that both these populations of neutrophils together with red pulp and marginal zone macrophages contribute to *S. pneumoniae* clearance (5). In the context of systemic *E. coli* infection the spleen also plays a critical role in host defense, as splenectomy leads to overwhelming sepsis in ~13% of patients (6–8). At a cellular level, marginal zone (MZ) macrophages have been shown to play a critical role in the capture of this pathogen (9), but the role of splenic neutrophils in this response has not been fully explored.

In this study we have used a combination of IVM, flow cytometry and precision cut spleen slices (PCSS) to explore the dynamic response of neutrophils in the blood, bone marrow, lung and spleen simultaneously both *in* and *ex vivo* in the first hour following a systemic challenge of heat-inactivated *E. coli* gaining insight into the temporal response of neutrophils in different tissues at this critical time point. We show that the capture of pathogens in the lung is initially mediated by both intravascular ‘resident’ neutrophils and endothelial cells and followed by the accumulation of circulating neutrophils leading to the formation of large clusters of neutrophils in the microvessels. In the spleen MZ macrophages bind and retain *E. coli* in the MZ, while splenic neutrophils migrate from the red pulp to the MZ where they form clusters around the pathogens. During this process, splenic levels of CXCL12 and ICAM-1 are decreased, while CXCL1, CXCL2, and IL-1β are up-regulated supporting changes in neutrophil dynamics. The CD29 (β_1_)-integrin is critical for neutrophil migration from the red pulp to the MZ, while the CXCL2-CXCR2 chemokine axis sustains neutrophil clustering. Thus, the results of this study show that lung and spleen resident neutrophils are pioneer cells in the response to systemic *E. coli* challenge.

## Materials and Methods

### Study Design

The primary objective of this study was to define the role of tissue resident neutrophils vs circulating neutrophils during systemic *E. coli* challenge. In all experiments, appropriate control groups were used. 6–8 weeks-old female mice were housed under the same environmental conditions, age-matched and randomly allocated to distinct experimental groups. The number of mice in each group, as well as the number of independent replicates, is stated in each figure legend.

### Mice

C57Bl/6J female mice between 6-8 weeks old were purchased from Charles River. All mice were housed in specific pathogen free conditions at Imperial College London. All experiments were carried out in accordance with the recommendations in the Guide for the Use of Laboratory Animals of Imperial College London. All animal procedures and care conformed strictly to the UK Home Office Guidelines under the Animals (Scientific Procedures) Act 1986, and the protocols were approved by the Home Office of Great Britain.

### Reagents

1x10^7^ Molecular Probes^®^ Bioparticles^®^ are fluorescently labelled with Fluorescein (FITC), Ex/Em (~494/518 nm), heat- or chemically inactivated *E. coli* (K-12 strain) were resuspended in 50μl of PBS and used i.v. as challenge. Blocking mAbs used were all from BioLegend: CD29-integrin blocking Ab (clone HMβ1-1), ICAM-1 (clone YN1/1.7.4). CXCR2 antagonist was from TOCRIS (SB225002); anti-CXCL1 and anti-CXCL2 and IgG were from (R&D Systems). Macrophages were depleted *via* i.p. injection of Standard Macrophage Depletion Kit (Clodronate^®^, Encapsome^®^) and control liposome from Encapsula 24h before *E. coli* i.v. injection. Neutrophil were depleted *via* i.p. injection of 100µg/mouse of anti-Ly6G mAb (clone 1A8) or 100µg/mouse of IgG2a (clone 2A3) 24h before *E. coli* i.v. injection.

### Tissue Preparation

Blood was collected in EDTA 2mM (Thermofisher) coated syringes by cardiac puncture under terminal anesthesia with Sodium-Pentobarbital. Spleens were collected and homogenized mechanically through a 70 μm filter in RPMI medium + 10% fetal bovine serum (FBS); BM cells were harvested from the femur by flushing the bone RPMI medium + 10% FBS. RBC lysis of the blood was carried out and samples were centrifuged at 1200 RPM for 5 min at 4˚C.

### Flow Cytometry

Single cell suspension from blood, spleen and BM were stained with Live/Dead near-IR stain (Life Technologies) and Fc-Receptors block (using clone 93, BioLegend). Cell suspensions were incubated with directly conjugated fluorescent antibodies for 30 min at 4°C. The following Abs were used: Ly6G (clone 1A8), CD45 (clone 30-F11), CD11b (clone M1/70), CD3e (clone 17 A2), CD19 (clone 6D5), Ter119 (clone TER-119), CD62L (clone MEL-14), CXCR4 (clone2B11), ICAM-1 (clone YN1/1.7.4) and CXCR2 (SA044G4) Acquisition was performed on BD Fortessa using FacsDiva software (BD Bioscience) with further analysis by FlowJo software (BD Bioscience). CD3e (clone 17 A2), CD19 (clone 6D5), Ter119 (clone TER-119) were used as a dulp gate for T cells, B cells and red blood cells respectively.

### Lung Intravital Microscopy (L-IVM)

This method was first described in (3) with modifications (10). Imaging was performed on an upright Leica SP5 confocal microscope using a 25x 0.95na water immersion objective. Neutrophils were labelled with 2-4μg/mouse of anti-Ly6G (clone 1A8)-PE, the vasculature was labelled with anti-CD31 (clone 390)-488, fluorescent Abs were injected i.v. in a maximal volume of 50 μl.

Blocking Abs or antagonists were i.v. injected while imaging continued non-stop until 1h after treatment. At the end of the imaging session, mice were humanely killed by anesthetic overdose (Sodium-Pentobarbital) and blood was collected by cardiac puncture and lung and spleen were harvested. Control mice were i.v. injected with sterile PBS or IgG Abs.

### Spleen Intravital Microscopy (S-IVM)

This method is described in (11) with modifications. The spleens of live mice were imaged under non-recovery, terminal anesthesia. Anesthesia was induced with medetomidine/ketamine combo i.p. and maintained with the alternating s.c. injections of either 50 mg/kg ketamine alone or in combination with 0.125 mg/kg medetomidine at predefined timepoints. Mice were placed in the right lateral decubitus position and a small section of hair was removed from the left flank. A 5-8mm abdominal incision on the left flank above the spleen was used to expose the surface of the spleen, which was mechanically stabilized with a gentle vacuum using the coverslip vacuum chamber used for lung IVM.

Imaging was performed on an upright Leica SP5 confocal microscope using a 25x 0.95na water immersion objective. Images were acquired in 3 z-slices 5 μm apart. Neutrophils were labelled with 2-4μg/mouse of anti-Ly6G (clone 1A8)-PE, the vasculature was labelled with anti-CD31 (clone 390)-Alexa488, and MZ macrophages were labelled with 2-4μg/mouse of anti-CD169-Alexa647. Fluorescent Abs were injected i.v. in a maximal volume of 50 μl.

### Precision Cut Lung Slices (PCLS)

To enhance Ab staining, mice were i.v. injected with 2-3μg of anti-Ly6G-PE (clone 1A8) 10-15 min prior organ collection. Mice were culled by i.p. injection of Sodium-Pentobarbital, then a small incision was made in the trachea and a cannula was inserted. 1ml of 2% low-melting point agarose was instilled through the cannula. Mice were placed on ice until the agarose set, lungs were collected en-block and fixed in 4% formaldehyde (v/v in PBS) (Thermofisher, UK) overnight. Lungs were sliced at 300 μm thick sections on a vibrating microtome.

### Precision Cut Spleen Splices (PCSS)

To enhance Ab staining, mice were i.v. injected with 2-3μg of anti-Ly6G (clone 1A8) and 2-3μg of anti-CD169 (clone 3D6.112) 10-15 min prior organ collection. As required, mice were i.v. injected with *E.coli* particles for the required time and then culled by i.p. injection of Sodium-Pentobarbital, an incision on the left flank was made and spleen was collected and kept live in HBSS+ 5% Hepes or fixed in 4% formaldehyde (v/v in PBS) (Thermofisher, UK) O/N. Spleens were sliced at 250-200 μm thick sections respectively on a vibrating microtome, using a protocol adapted from (12). Live PCSS were cultured ex vivo in incubator at 37˚C 5% CO_2_ for 1h and 2h in RPMI medium + 5% FBS. As internal control, one slice was not incubated and fixed.

### Cell Tracking

L-IVM and S-IVM 3D time-series in.lif format were analyzed using Imaris software (Bitplane, Oxford Instruments). The videos were cropped in time to analyze neutrophil speed in 60 frames before and 60 frames after 60 min of *E. coli* challenge. Neutrophil tracking was performed automatically on Ly6G-positive cells segmented as spots. Neutrophil XYZ location data were exported and mean track speed was plotted.

### ELISA

Supernatants from spleen, BM and blood serum were used for ELISA. The following DuoSet ELISAs (R&D Systems) were used: Mouse CXCL1 (DY493), Mouse CXCL2 (DY492), Mouse IL-1β (DY401), and Mouse CXCL12 (DY460).

### Statistical Analysis

Statistical analysis was performed using GraphPad Prism 8 (GraphPad Software, Inc). A p-value of less than 0.05 was considered significant: P<0.05 *, P<0.01 **, P<0.001 ***, P<0.0001 ****, NS=not significant. Statistical tests used are mentioned in the figure legends.

### Data Sharing Statement

For original data, please contact k.de-filippo@imperial.ac.uk.

## Results

### Contribution of Circulating Neutrophils in the Capture of Heat-Inactivated *E. coli* and Later Neutrophils Mobilization From the Bone Marrow

**Figure 1:**
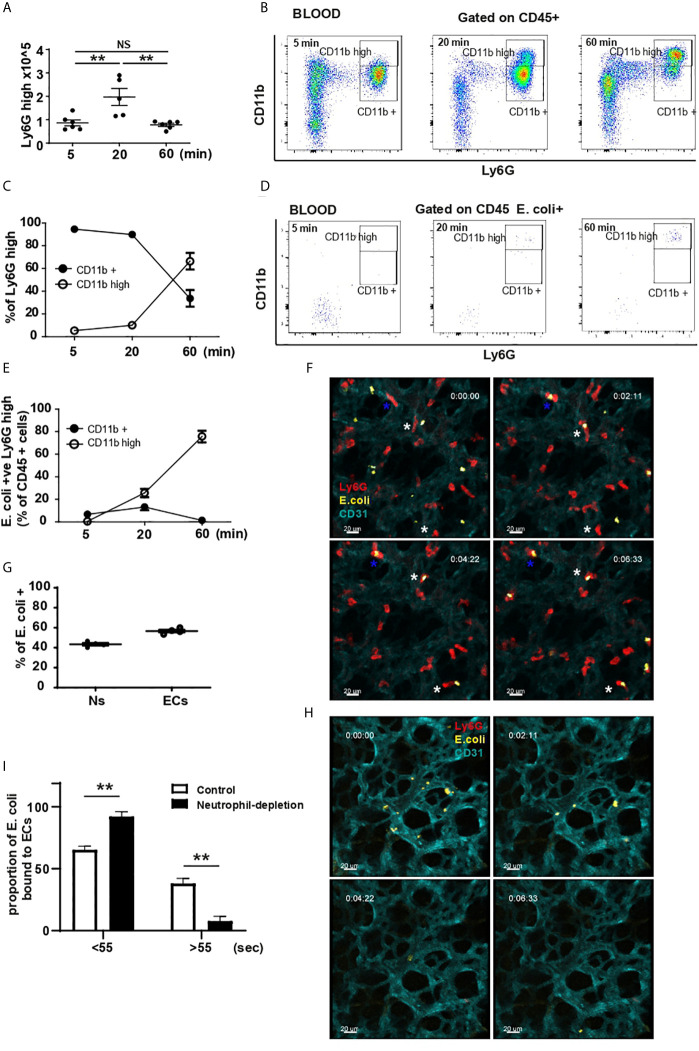
Lung marginated and circulating neutrophils activation and contribution to *E. coli* capture. **(A)** Total number of neutrophils by flow cytometry 5, 20 and 60 min after i.v. injection of *E. coli* (N=5-6 mice/timepoint), one-way ANOVA, Tukey’s multiple comparison test, three independent experiments. **(B)** FACS analysis of circulating neutrophils 5, 20 and 60 min after i.v. injection of *E. coli*, representative of two independent experiments. **(C)** % of Ly6G^high^ CD11b^+^ and Ly6G^high^ CD11b^high^ neutrophils was calculated 5, 20 and 60 min post i.v. injection of heat-inactivated *E. coli*, (N=4 mice/timepoint). **(D)** Flow cytometry analysis of *E. coli*-positive circulating cells 5, 20 and 60 min after i.v. injection of *E. coli*, representative of two independent experiments. **(E)** *E. coli*-positive Ly6G^high^ CD11b^+^ and Ly6G^high^ CD11b^high^ neutrophils as % of CD45+ cells was calculated 5, 20 and 60 min post i.v. injection of heat-inactivated *E. coli*, (N=4 mice/timepoint). **(F)** Sequential frames from video 1, white asterisk shows neutrophil (red) migration toward heat-inactivated *E. coli* captured by ECs (cyan); blue asterisk shows a neutrophil that captured heat-killed *E. coli* (yellow) directly from the circulation. Timing represents time from start of imaging. Scale bar 20μm. **(G)** Quantification of EC and neutrophil (Ns) contribution in capturing *E. coli* from the circulation (N=3 mice). **(H)** Sequential frames from video 2, showing ECs inability to retain *E. coli* particles. Timing represents time from start of imaging. Scale bar 20μm. **(I)** Proportion of *E. coli* particles bound to ECs over number of frames in the presence or depletion of neutrophils (N=3 mice/group), one-way ANOVA, Sidak’s multiple comparison test, three independent experiments. P < 0.01 **.

To investigate the effects of intravascular (i.v.) injection of heat-inactivated *E. coli* on neutrophil numbers, Ly6G^high^ neutrophils (**Supplementary Figure 1A**) were counted in the blood (**Figure 1A**) and bone marrow (BM) (**Supplementary Figure 1B**) at 5, 20 and 60 min post injection of AlexaFluor 488 conjugated *E. coli* bio-particles in mice. We observed a significant increase in circulating neutrophil numbers 20 min after challenge that returned to basal levels 60 min post *E. coli* injection (**Figure 1A**).

There was a concomitant decrease in bone marrow numbers of Ly6G^high^ neutrophils that was significant 60 min post injection (**Supplementary Figure 1B**). Following challenge, a subpopulation of CD11b^high^ neutrophils appeared in the blood 20 min post challenge and increased by 60 min (**Figures 1B, C**). Analysis of *E. coli* phagocytosis of CD45^+^ cells showed that by 20 min the predominant *E. coli*-positive cells were neutrophils, specifically CD11b^high^ Ly6G^high^ subpopulation (**Figures 1D, E** and **Supplementary Figure 1C**). Concomitant with the increase of CD11b on neutrophils, the shedding of CD62-L was detectable in both CD11b^+^ and CD11b^high^ Ly6G^high^ populations of neutrophils (**Supplementary Figure 1D**). However, it was more pronounced on Ly6G^high^ CD11b^high^ cells consistent with a more inflammatory phenotype (4, 13). While a substantial number of Ly6G^high^ neutrophils remain in the bone marrow at 60 min post challenge (**Supplementary Figure 1B**), very few of these cells were associated with *E. coli* (Figure **Supplementary Figure 1E**). Taken together, these data indicate a specific uptake of *E. coli* by circulating CD11b^high^ neutrophils from 20 min that continues to rise to 60 min post challenge. The reduction in numbers of neutrophils in the bone marrow suggests a potential source of the increased circulating neutrophils. Of note, between 20 and 60 min after *E. coli* challenge, the number of neutrophils in the BM continued to decrease, but this was not mirrored by an increase in neutrophils in the blood, suggesting that mobilized neutrophils were potentially accumulating in tissues.

### *E. coli* Is Rapidly Captured by Both the Lung Microvasculature Endothelium and Marginated Neutrophils

Applying IVM to the lung (L-IVM) we studied the *in vivo* dynamic behavior and early contribution of lung ‘resident’ neutrophils and the microvasculature endothelium to *E. coli* recognition and capture. Within the first seconds to minutes after challenge, lung endothelial cells (ECs) and marginated neutrophils captured *E. coli* directly from the circulation (**Figure 1F** blue * and video 1). Quantification revealed that of the *E. coli* particles retained in the lung, ~40% were initially captured by neutrophils (Ns) while ~60% were captured by ECs (**Figure 1G**). L-IVM also revealed that marginated neutrophils subsequently migrated towards the heat-inactivated *E. coli* adherent to ECs and appeared to bind/or ingest them (**Figure 1F** white *, video 1).

Neutrophil-depletion by i.p. injection of high dose anti-Ly6G mAb revealed that while ECs were still able to capture *E. coli* within the first seconds post challenge (**Figure 1H**, video 2), they failed to retain them efficiently when neutrophils were depleted (**Figure 1H**, video 2). In the presence of neutrophils, ~38% ECs were able to retain the particles beyond 55 seconds, however, when neutrophils were depleted, only ~8% of ECs retained the pathogen (**Figure 1I**), suggesting a profound cooperation between these two cell types to efficiently retain pathogens within the lung microvasculature. These results suggest that lung marginated neutrophils are pioneer cells and key contributors in the initial removal of circulating pathogens *via* two mechanisms: neutrophils directly recognize and capture circulating *E. coli* and neutrophils modulate ECs to prolong their retention of circulating pathogens.

### *E. coli* Positive Neutrophils Forming Clusters Within Lung Microvessels Express CD11b

**Figure 2:**
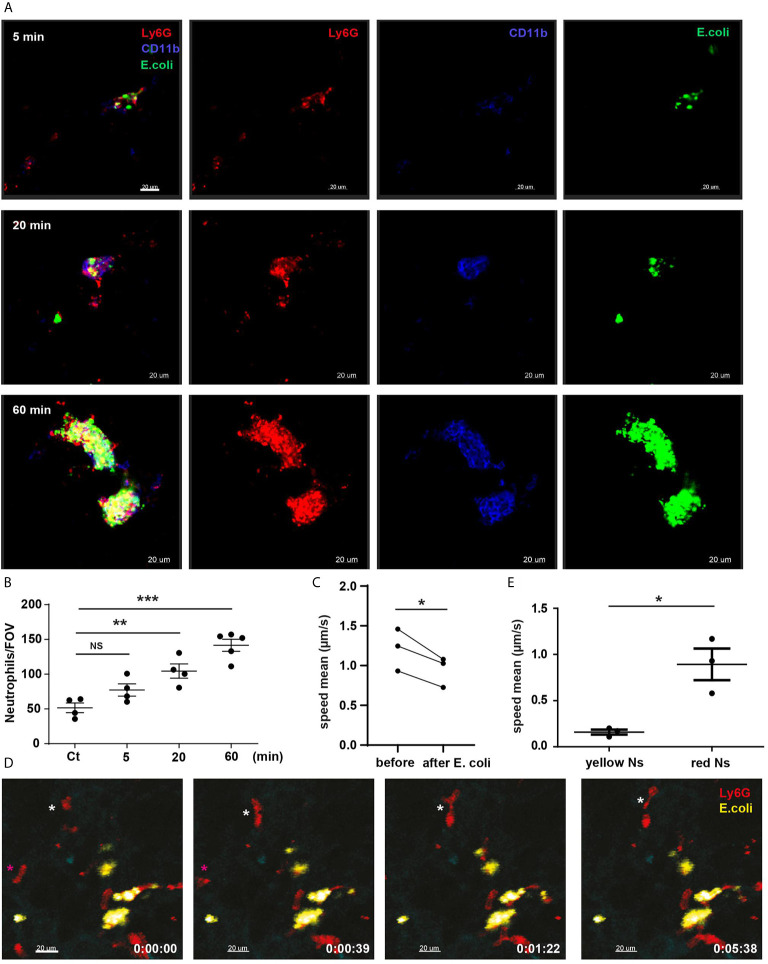
Lung marginated neutrophils are CD11b-positive and form clusters enriched in *E. coli*. **(A)** PCLS after *E. coli* challenge 5, 20 and 60 min. Scale bar 20μm. **(B)** Total number of neutrophils/FOV counted in PCLS after PBS (Ct) for 60 min or *E. coli* challenge for 5, 20 and 60 min (N=4-5 mice/timepoint), (10 FOV/mouse), one-way ANOVA, Tukey’s multiple comparison test, two independent experiments. **(C)** L-IVM analyzed for neutrophil speed mean comparing before and 60 min after *E. coli* challenge (N=3 mice), paired *t-test*. **(D)** Sequential frames from video 3, *E. coli*-positive neutrophils (yellow Ns) are stationary, while *E. coli*-negative neutrophils (red Ns) are highly motile (white and magenta asterisks). Scale bar 20μm. P < 0.05 *, P < 0.01 **, P < 0.001 ***, NS, not significant. **(E)** L-IVM analyzed for neutrophil speed mean comparing *E. coli*-positive neutrophils (yellow) and *E. coli*-negative neutrophils (red) 60 min after *E. coli* challenge (N=3 mice), *t-test*.

Precision cut lung slices (PCLS) harvested 5, 20 and 60 min after challenge, revealed that *E. coli*-positive marginated neutrophils also expressed CD11b (**Figure 2A**). A significant increase in the number of intravascular marginated neutrophils per field of view (FOV) was apparent at 20 min and increased further by 60 min post challenge when compared to PBS (Ct) (**Figures 2A, B**).

Analysis of L-IVM by comparing mean neutrophil speed in the same FOV before and 60 min after systemic *E. coli* revealed that intravascular ‘retained’ lung neutrophils exhibited a markedly reduced migratory speed after challenge (**Figure 2C**, video 3). *E. coli*-positive marginated neutrophils were static (yellow neutrophils, **Figure 2D**, video 3, **Figure 2E**) while *E. coli*-negative marginated neutrophils were motile (red neutrophils, **Figure 2D** magenta and white *, video 3, **Figure 2E**). Our data show that marginated neutrophils migrate towards *E. coli* particles captured by ECs and rapidly aggregate in clusters enriched in *E. coli*. Thus, the lungs appear to be an important site for the collection of circulating *E. coli* and marginated neutrophils are critical in promoting this process.

### Splenic Resident Neutrophils Are Sufficient to Build a Response to Systemic *E. Coli*

While the highly vascularized nature of the lung makes it a prominent site of neutrophil interaction with pathogens, the spleen also plays a key, but distinct role in coordinating immunological responses to blood-borne pathogens (14, 15). Blood percolates through the spleen at a reduced velocity allowing pathogens present in the circulation to be filtered (14, 15). In a model of *S. pneumoniae* challenge, it has been shown that both red pulp macrophages and neutrophils are essential for pathogen recognition and clearance (5). In contrast, immunohistochemistry data indicated that most *E. coli* are captured by MZ macrophages (9).

**Figure 3:**
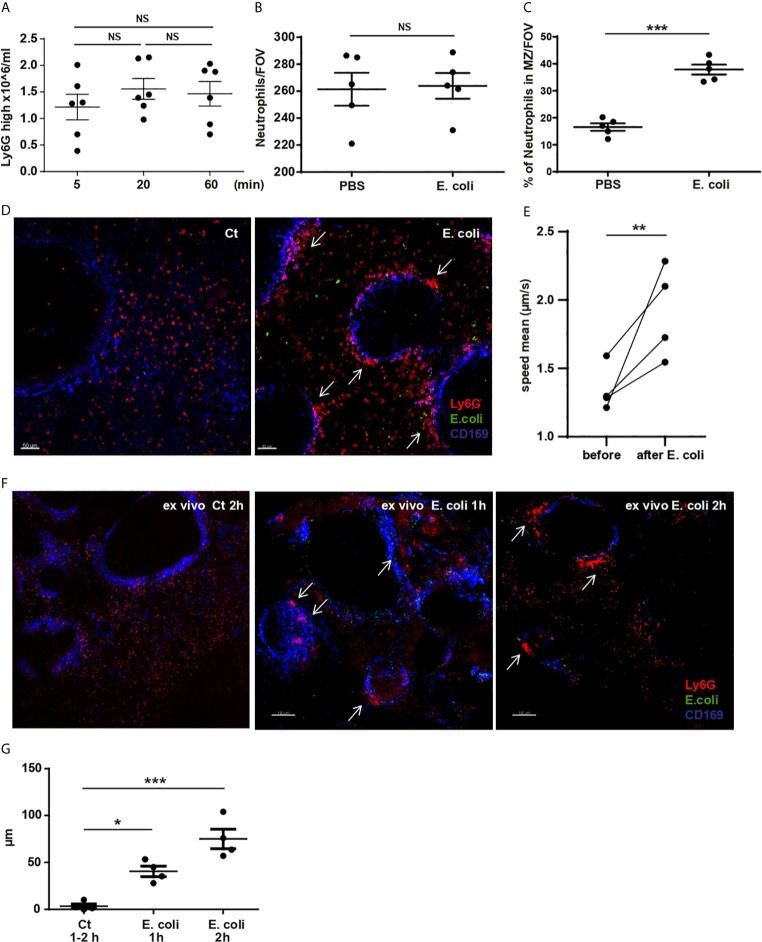
Splenic resident neutrophil activation and redistributing within the spleen after systemic *E. coli* challenge. **(A)** Total number of splenic neutrophils 5, 20 and 60 min after i.v. injection of *E. coli*, evaluated by flow cytometry, one-way ANOVA, Tukey’s multiple comparison test, three independent experiments, (N=6 mice/timepoint). **(B)** Total number of neutrophils/FOV counted in PCSS after PBS or challenge with *E. coli* for 60 min, unpaired *t-test* (N=4 mice/group), (10 FOV/mouse). **(C)** % of splenic neutrophils located in the MZ/FOV counted in PCSS after PBS or *E. coli* challenge for 60 min, unpaired *t-test*, (N=4 mice/group), (10 FOV/mouse). **(D)** PCSS image after PBS or challenged with *E. coli* for 60 min. Scale bar 50μm. Arrows point to neutrophil clusters in the MZ. **(E)** S-IVM analyzed for neutrophil mean speed comparing before and 60 min after *E. coli* challenge, paired *t-test*, (N=4 mice). **(F)** 3D *ex-vivo* culture system of living PCSS for 1h and 2h, pre-challenged with systemic *E. coli* for 5min or PBS (Ct). Scale bar 150μm. P < 0.05 *, P < 0.01 **, P < 0.001 ***, NS=not significant. Arrows point to neutrophil clusters in the MZ. **(G)** Diameter of neutrophil clusters in 3D ex vivo culture system of living PCSS cultured for 1h or 2h, pre-challenged with systemic E. coli for 5 min or PBS, one-way ANOVA, Tukey’s multiple comparison test, four independent experiments, (N=4 mice/timepoint).

PCSS showed that while pathogens were clearly concentrated in the MZ within the first 5 min of challenge, they were also present in the red pulp (**Supplementary Figures 2A, B**). In contrast to the lungs, 60 min post challenge we observed no change in the absolute number of splenic neutrophils, as measured by flow cytometry (**Figure 3A**) or by quantification of neutrophils/FOV in PCSS (**Figure 3B**). However, we noticed a substantial change in the localization of splenic neutrophils after challenge, with neutrophils relocating to the MZ (**Figures 3C, D**) and clustering in close proximity to CD169-positive macrophages (**Figure 3D** arrows). S-IVM revealed that the migratory speed of splenic neutrophils significantly increased following systemic challenge (**Figure 3E**) migrating towards *E. coli* (video 4) and forming clusters by 60 min post challenge (**Figure 3D** arrows), thus, following similar kinetics for cluster formation as seen in the lung microvessels.

To further verify that circulating neutrophils were not accumulating in the spleen post *E. coli*, we established a 3D *ex vivo* culture system of living spleen slices. 5 min after systemic challenge, the spleen was harvested, sliced, and incubated in a 3D *ex vivo* culture system for 1h or 2h (**Figure 3F**). Using this system, we showed the relocation of splenic red pulp neutrophils to the MZ and the formation of clusters (**Figure 3F** arrows and **Figure 3G**) that enlarged over time even in the absence of circulating neutrophils.

**Figure 4:**
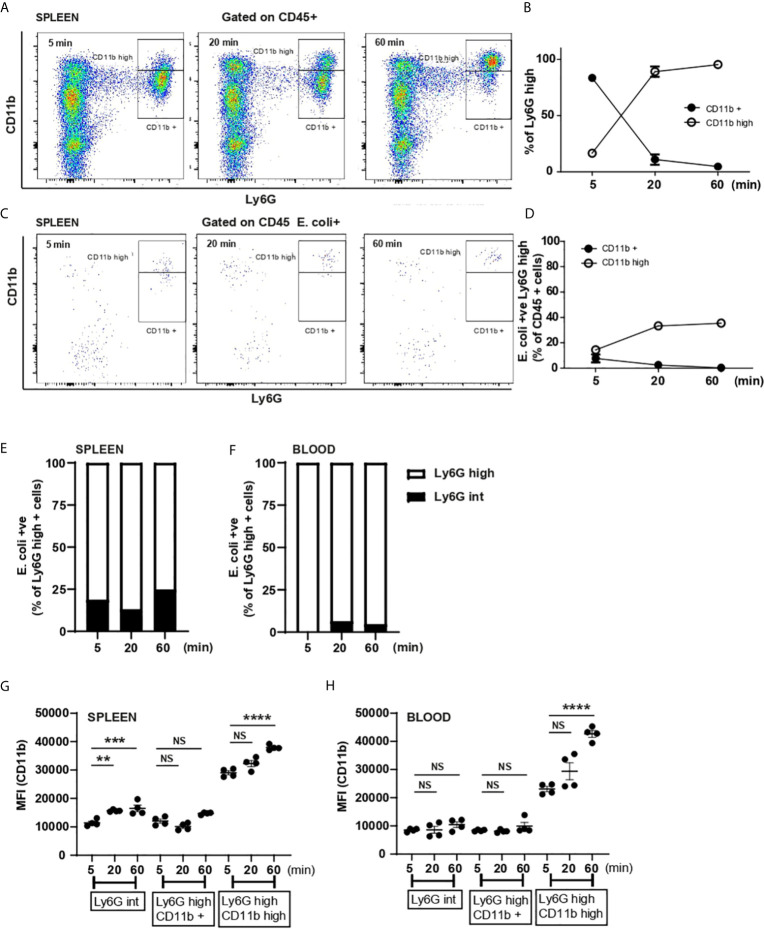
Splenic neutrophils activation and Ly6G^int^ and Ly6G^high^ contribution to systemic *E*. *coli* recognition. **(A)** Flow cytometry analysis of splenic neutrophils 5, 20 and 60 min after i.v. injection of *E. coli*, representative of two independent experiments. **(B)** % of Ly6G^high^ CD11b^+^ and Ly6G^high^ CD11b^high^ neutrophils was calculated 5, 20 and 60 min post i.v. injection of heat-inactivated *E. coli*, (N=4 mice/timepoint). **(C)** Flow cytometry analysis of *E. coli*-positive splenic cells 5, 20 and 60 min after i.v. injection of *E. coli*, representative of two independent experiments. **(D)** *E. coli*-positive neutrophils as % of CD45+ cells were calculated 5, 20 and 60 min post i.v. injection of heat-killed *E. coli*, (N=4 mice/timepoint). **(E)** % of Ly6G^int^ and Ly6G^high^ of total E. coli-positive splenic neutrophils at 5, 20 and 60 min post challenge, (N=4 mice/timepoint). **(F)** % of Ly6G^int^ and Ly6G^high^ of total E. coli-positive circulating neutrophils at 5, 20 and 60 min post challenge, (N=4 mice/timepoint). **(G)** Median fluorescent intensity (MFI) (anti-CD11b fluorophore) on splenic Ly6G^int^, Ly6G^high^ CD11b^+^ and Ly6G^high^ CD11b^high^, one-way ANOVA, Tukey’s multiple comparison test, two independent experiments, (N=4 mice/group). **(H)** Median fluorescent intensity (MFI) (anti-CD11b fluorophore) expression on circulating Ly6G^int^, Ly6G^high^ CD11b^+^ and Ly6G^high^ CD11b^high^, one-way ANOVA, Tukey’s multiple comparison test, two independent experiments, (N=4 mice/group). P < 0.01 **, P < 0.001 ***, P < 0.0001 ****, NS, non significant.

The uptake of *E. coli* by splenic resident neutrophils was also associated with an increase in CD11b surface expression, however, the dynamics were faster compared to circulating neutrophils. Thus ~15% of Ly6G^high^ splenic neutrophils were already CD11b^high^ 5 min post *E. coli* challenge and the % increased with time (**Figures 4A, B**). ~6.05 ± 0.72 (N=4) % of Ly6G^high^ splenic neutrophil were CD11b^high^ during homeostasis (**Supplementary Figure 2C**). 5 min after challenge, similar % of CD11b^+^ and CD11b^high^ splenic neutrophils were *E. coli*-positive, but by 20 min already ~40% were CD11b^high^ (**Figures 4C, D**). Other splenic non-neutrophil cells were able to capture *E. coli* (**Figure 4C**); marginal zone macrophages have a high phagocytic affinity for *E. coli* (9). PCSS clearly showed that 5 min post challenge ~80% of pathogens were found in the MZ of the spleen (**Supplementary 2B**). Consistent with the activated neutrophil phenotype, the shedding of CD62-L was detectable in both CD11b^+^ and CD11b^high^ Ly6G^high^ splenic resident neutrophils after challenge (**Supplementary Figure 2D**).

### Ly6G^int^ Marginally Contribute to the Capture of *E. coli*

Deniset et al. (5) showed that Ly6G^int^ neutrophils present in the spleen represented an immature phenotype that could mature and contribute to *S. pneumoniae* uptake (5). Therefore, we extended our analysis to assess the relative contribution of Ly6G^int^ and Ly6G^high^ neutrophils to *E. coli* capture and found that ~ 20% of splenic neutrophils capturing *E. coli* were Ly6G^int^ (**Figure 4E**). In contrast when we analyzed the circulating Ly6G^int^ population, we found that they did not participate in pathogen recognition and elimination from the circulation (**Figure 4F**). Investigating expression of CD11b on these different populations of neutrophils we saw a fast up-regulation of CD11b on splenic Ly6G^int^ neutrophils (**Figure 4G**) while the level of expression by 60 min did not increase on blood Ly6G^int^ neutrophils (**Figure 4H**). However, the upregulation of CD11b on splenic Ly6G^int^ (**Figure 4G**) was very modest compared with that on Ly6G^high^ CD11b^high^ neutrophils (**Figures 4G, H**). This led us to conclude that splenic immature neutrophils only marginally participate to *E. coli* clearance.

### MZ Macrophages Contribute to *E. coli* Retention Within the MZ but Play No Role in Neutrophil-*E. coli* Interaction

The spleen harbors several subsets of macrophages with specific locations and functions (16). CD169-positive macrophages occupy the MZ and are the primary line of protection against several blood-borne pathogens including *E. coli* (9). Mice lacking CD169-positive macrophages showed a significant increase in *E. coli* growth in the spleen (17).

**Figure 5:**
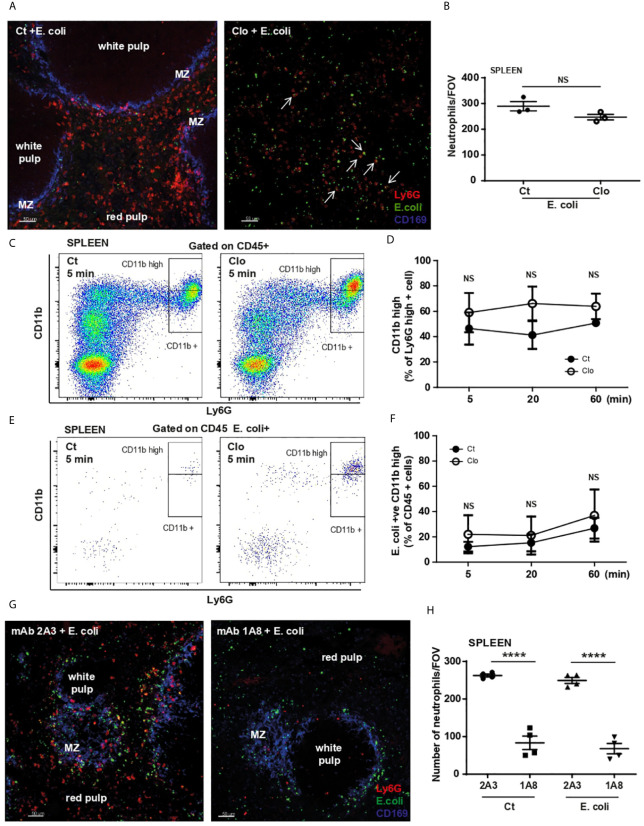
MZ macrophages and splenic neutrophil functions during *E. Coli* challenge. **(A)** PCSS image of liposome-control (Ct) and liposome-clodronate (Clo) pre-treated mice challenged for 60 min with systemic *E. coli*. Scale bar 50μm. Arrows point to some neutrophils that co-localize with *E. coli*. **(B)** Total number of neutrophils/FOV counted in PCSS of liposome-control and liposome-clodronate pre-treated mice challenged for 60 min with systemic *E. coli*, unpaired t-test (N=3 mice/treatment), (10 FOV/mouse). **(C)** Flow cytometry analysis of splenic neutrophils 5 min after i.v. injection of *E. coli*, representative of two independent experiments. **(D)** % of Ly6G^high^ CD11b^high^ neutrophils of liposome-control (Ct) and liposome-clodronate (Clo) pre-treated mice challenged for 5, 20, and 60 min with systemic *E. coli*, (N=4 mice/timepoint). **(E)** Flow cytometry analysis of *E. coli*-positive splenic cells 5 min after i.v. injection of *E. coli*, representative of two independent experiments. **(F)** *E. coli*-positive Ly6G^high^ CD11b^high^ neutrophils as % of CD45+ cells were calculated 5, 20 and 60 min post i.v. injection of heat-inactivated *E. coli*, (N=4 mice/timepoint). **(G)** PCSS image of 2A3 mAb and 1A8 mAb pre-treated mice challenged for 60 min with systemic *E. coli*. Scale bar 50μm. **(H)** Total number of neutrophils/FOV counted in PCSS of 100 µg of 2A3 mAb and 100 µg of 1A8 mAb pre-treated mice challenged for 60 min with systemic PBS or *E. coli*. (N=4 mice/group), (10 FOV/mouse). P < 0.0001 ****, NS, non significant.

To investigate the interplay between MZ macrophages and red pulp resident neutrophils in the capture of heat-inactivated *E. coli*, a low dose of clodronate liposomes was used to deplete macrophages in the MZ (5). PCSS of macrophage-depleted mice, harvested 60 min post challenge, confirmed the depletion of CD169-positive macrophages and showed that, in their absence *E. coli* particles were scattered throughout the spleen (**Figure 5A**) thus confirming the importance of CD169-positive macrophages in the retention of pathogens within the MZ. Clodronate treatment did not significantly influence the number of splenic neutrophils/FOV (**Figure 5B**) and neutrophils were still found to co-localize with *E. coli* particles (**Figure 5A** arrows). Moreover, their ability to up-regulate CD11b (**Figures 5C, D**) or to phagocyte *E. coli* (**Figures 5E, F**) was not affected by the absence of MZ macrophages. However, in the absence of MZ macrophages the neutrophils failed to organize in clusters (**Figure 5A**), suggesting that macrophages drive this process by attracting neutrophils to the MZ.

Neutrophil-depletion revealed that location of *E. coli* particles within the spleen was not compromised (**Figure 5G**). However, depletion of splenic neutrophils was not complete (**Figure 5G**), in accordance with Deniset et al. (5) where the authors showed the remaining cells were Ly6G^int^ (5). Indeed, quantification of neutrophils/FOV revealed that 1/3 of splenic neutrophils remained after a high dose of mAb Ly6G (**Figure 5H**). However, in the neutrophil-depleted slices, the remaining Ly6G^int^ neutrophils do not form clusters in the spleen (**Figure 5G**). In contrast, in the lung, high dose of mAb Ly6G caused a total depletion of neutrophils (**Supplementary Figure 2E**). These data suggest while uptake of *E. coli* by splenic neutrophils is independent of MZ macrophages, these cells are critical in orchestrating the compartmentalization of *E. coli* in the spleen and drive neutrophil relocation and clustering.

### CXCR2-CXCL2 Axis Is Important for Cluster Enlargement

**Figure 6:**
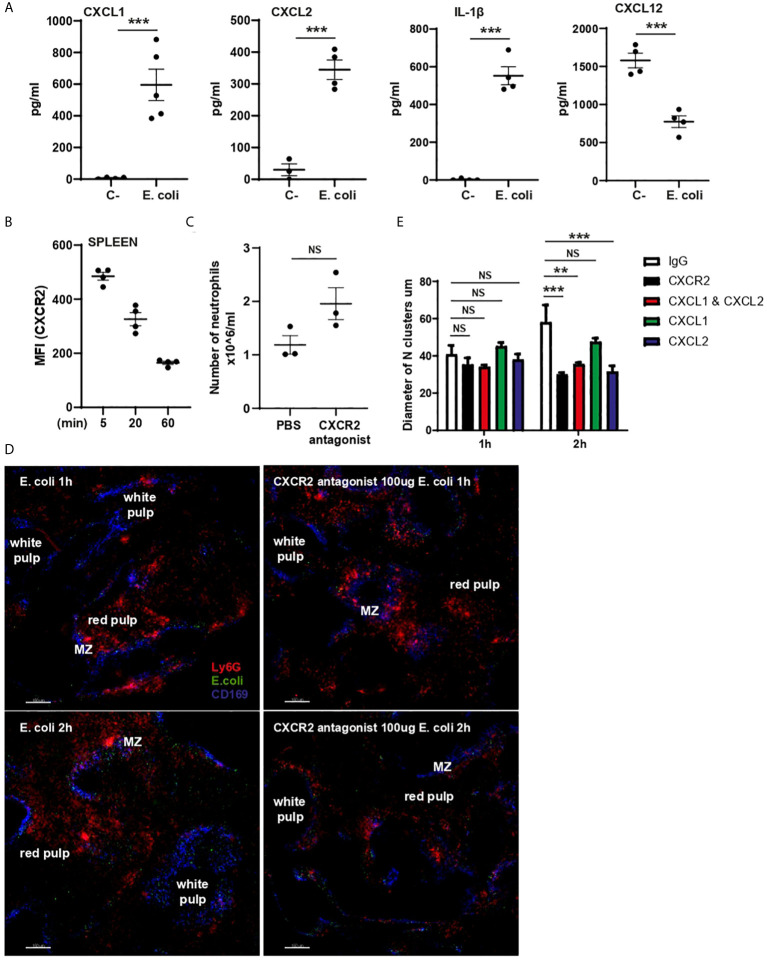
CXCL1, CXCL2, IL-1β and CXCL12 expression in splenic supernatant and CXCR2 role in cluster enlargement. **(A)** CXCL1, CXCL2, IL-1β and CXCL12 ELISAs of splenic supernatant of PBS **(C-)** or *E. coli* treated mice (N=4-5). **(B)** MFI (anti-CXCR2 fluorophore) expression on splenic neutrophils 5, 20 and 60 min after i.v. injection of *E. coli*, (N=4 mice/treatment). **(C)** Total number of splenic neutrophils of mice treated for 60 min with PBS or CXCR2 antagonist, unpaired t-test (N=3 mice/treatment). **(D)** 3D *ex-vivo* culture system of living PCSS over time pre-treated with PBS or CXCR2 antagonist and challenged with systemic *E. coli* for 5min. Scale bar 150µm. **(E)** Quantification of the diameter of neutrophil clusters in 3D ex-vivo culture of living PCSS over time pre-treated with IgG, CXCR2 antagonist, CXCL1 mAb, CXCL2mAb, and CXCL1 and CXCL2 mAb over time, one-way ANOVA, Tukey’s multiple comparison test, two independent experiments, (N=3 time/treatment). P < 0.01 **, P < 0.001 ***, NS, non significant.

Splenic levels of CXCL1, CXCL2, and IL-1β significantly increased post challenge, while CXCL12 levels were significantly reduced (**Figure 6A**). We observed a decrease in the expression level of CXCR2 on splenic Ly6G^high^ neutrophils treated with *E. coli* (**Figure 6B**). To investigate whether the CXCR2/CXCL1/CXCL2 chemokine axis played a role in the process of neutrophil migration and/or cluster formation following challenge, mice were pre-treated i.p. with a CXCR2 antagonist (18). Blocking CXCR2 had no effect on the absolute number of splenic neutrophils (**Figure 6C**). To examine the effect of CXCR2 blockade on neutrophil migration and clustering, spleens were harvested from mice, pre-treated with or without CXCR2 antagonist, 5 min after *E. coli* challenge and then PCSS incubated in 3D tissue culture *ex vivo* for up to 2h. Pre-treatment with the CXCR2 antagonist did not impaired cluster formation 1h after challenge, however the formation of larger clusters was significantly inhibited (**Figures 6D, E**), suggesting a role for CXCR2 in cluster enlargement. Making use of mAb CXCL1 and/or CXCL2 we identified a specific role for CXCL2, but not CXCL1, in the enlargement of neutrophil clusters (**Figure 6E**).

Clodronate treatment reduced CXCL1 and CXCL2 generation in the spleen in response to challenge, indicating that CD169-positive macrophages partially contributed to chemokine production (**Supplementary Figure 2F**). Lack of CD169-positive macrophages almost totally abrogated the generation of splenic IL-1β but had no effect on splenic CXCL12 (**Supplementary Figure 2F**). Neutrophil-depletion showed a modest but significant down-regulation of CXCL2 and unexpected up-regulation of CXCL1, while levels of both IL-1β and CXCL12 were unaffected (**Supplementary Figure 2G**). These data indicate that in response to *E. coli* splenic macrophages generate IL-1β and both splenic macrophages and neutrophils contribute to the generation of CXCL2. While the level of CXCL12 was significantly reduced after challenge, there is no evidence that neutrophils or macrophages are involved in regulating the production of this chemokine.

### CD29 (β_1_) Integrin Plays a Role in Neutrophil Relocation Within the Spleen Following *E. coli* Challenge

**Figure 7:**
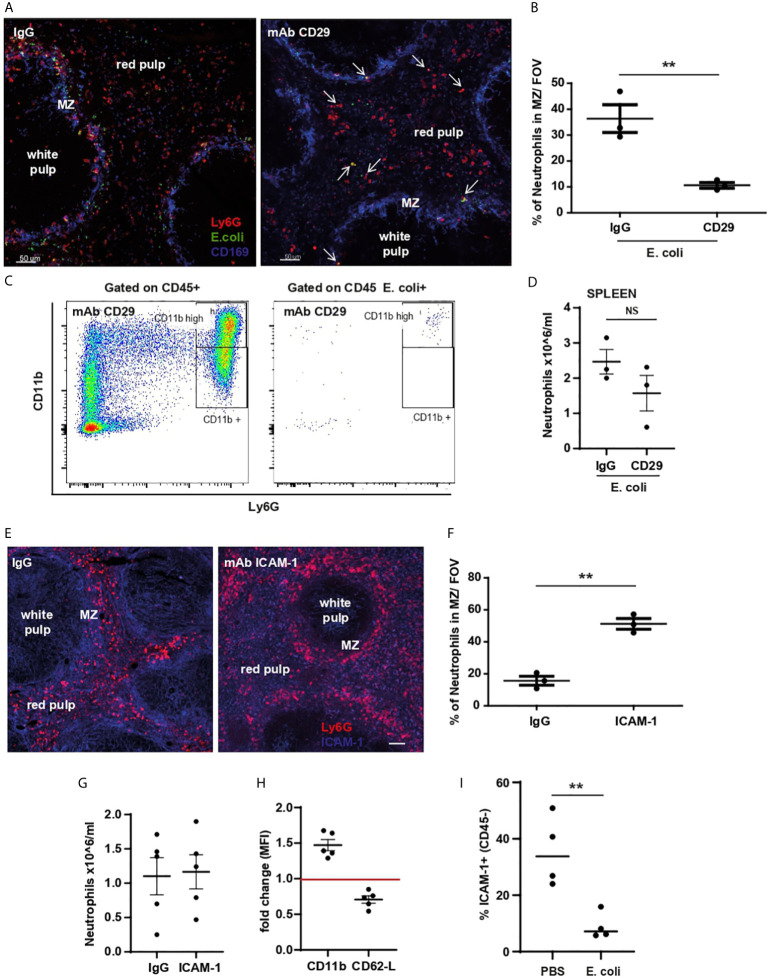
CD11b-, CD29-integrin and ICAM-1 role in splenic neutrophil retention, relocation, and clustering. **(A)** PCSS image of IgG and anti-CD29 mAb pre-treated mice challenged for 60 min with systemic *E. coli*. Scale bar 50μm. Arrows point to some neutrophils that co-localize with *E. coli* after anti-CD29 mAb treatment **(B)** % of splenic neutrophils in the MZ/FOV counted in PCSS of IgG and anti-CD29 mAb pre-treated mice challenged for 60 min with systemic *E. coli*, unpaired *t-test*, (N=3 mice/group). **(C)** Flow cytometry analysis of *E coli*-positive splenic neutrophils pre-treatment with CD29 mAb. **(D)** Quantification of splenic neutrophils 60 min after i.v. injection of *E. coli* pre-treated with anti-CD29 mAb, two independent experiments (N=3 mice/treatment). **(E)** PCSS image of IgG or anti-ICAM-1 mAb treated mice. Scale bar 50μm. **(F)** % of splenic neutrophils located in the MZ/FOV counted in PCSS after IgG or ICAM-1 mAb for 60 min, unpaired *t-test*, (N=3 mice/group). **(G)** Quantification of splenic neutrophils from mice treated for 60 min with i.v. IgG or ICAM-1 mAb, two independent experiments (N=5 mice/treatment). **(H)** MFI of anti-CD11b and anti-CD62L mAb of splenic neutrophils after anti-ICAM-1 mAb or IgG treatment expressed as fold change. **(I)** % of ICAM-1 expressed by CD45- splenocytes 60 min after i.v. injection of PBS or *E. coli*, representative of three experiments, unpaired t-test, (N=4 mice/treatment). P < 0.01 **, NS, non significant.

CD29 is highly expressed by neutrophils and supports their migration within tissues and/or cluster formation (19). A specific anti-CD29 blocking mAb was used to test whether CD29 was involved in the retention of neutrophils within the red pulp of the spleen, or their migration from red pulp to the MZ and clustering in response to *E. coli*. Pre-treatment with anti-CD29 mAb followed by *E. coli* challenge for 60 min impaired the relocation of splenic neutrophils in the red pulp to the MZ (**Figures 7A, B**). Blocking CD29 prior to challenge did not affect the up-regulation of CD11b or the *E. coli* capture by splenic neutrophils retained within the spleen 60 min post challenge (**Figure 7C**), but had a partial effect on the number of retained neutrophils (**Figure 7D**) suggesting CD29 may be required for neutrophil retention and is essential for migration to the MZ, not for capturing *E. coli*, nor for neutrophil retention within the spleen.

ICAM-1, a ligand for β_2_ integrins, is highly expressed on splenic CD45-negative stromal cells (20, 21). 60 min after administration of anti-ICAM-1 mAb, in the absence of pathogens, splenic neutrophils showed a dramatic displacement from the red pulp to the MZ (**Figures 7E, F**). While the number of splenic neutrophils did not change (**Figure 7G**), their surface expression of CD11b and CD62-L were significantly up- and down-regulated respectively (**Figure 7H**), suggesting an activated neutrophil phenotype. The % of ICAM-1 expression on splenic CD45-negative cells was reduced following *E. coli* treatment (**Figure 7I**). Taken together our data suggest that down regulation of ICAM-1 on stromal cells following *E. coli* challenge, releases neutrophils allowing them to migrate *via* CD29-integrin towards the MZ.

## Discussion

Systemic infections with *E. coli* account for 25-50% of all blood stream infections globally with a 20% mortality rate (22, 23). In our study, we show novel findings comparing critical but distinct sites of neutrophil surveillance in the lung and in the spleen after heat-inactivated *E. coli*. In response to challenge, neutrophils in the lung, spleen and blood capture *E. coli*, albeit with different kinetics, and take on an activated phenotype (CD11b^high^, CD62-L^low^). These data illustrate the dynamic nature of neutrophil responses. They support a scenario whereby pathogen stimulates the generation of cytokines or chemokines that stimulate neutrophil mobilization from the bone marrow, those neutrophils contribute to mopping up *E. coli* in the blood and rapidly concentrating it in the lung. Thus, the increase in circulating neutrophils is only observed transiently as these mobilized neutrophils are rapidly accumulating in the lung.

Pioneering work in 1987, by Lien et al. (23), first made use of fluorescent video microscopy to observe labelled neutrophils migrating in the sub-pleura pulmonary microcirculation through a window inserted into the chest cavity of dogs. In this study, the authors observed that under homeostasis neutrophils were making transient contacts with ECs and migrated within the pulmonary capillaries with a transit time ranging from 2 seconds to 20 min but had no engagement in arterioles or venules. Lung IVM is now a well-established technique that has been used by us and others to image murine neutrophil dynamics both under steady state and during infection (3, 4, 24). Three different neutrophil dynamic behaviors: tethering, crawling and firm adhesion, have been observed under homeostasis within the microcapillary of murine lungs (3) while 35% of neutrophils remained stationary/adherent (4). These studies suggest that there is a marginated pool of neutrophils in murine and canine lungs. It is unlikely that these marginated neutrophils are neutrophils that have become activated during tissue preparation as they are not clustering, a response we see with activated neutrophils and when the tissue is damaged.

In our study, we show that in the absence of ‘resident’ neutrophils, the lung microvasculature ECs failed to retain *E. coli* bio particles suggesting that neutrophil depletion does not interfere with the ability of the ECs to capture *E. coli* but marginated neutrophils and ECs act co-operatively to retain *E. coli*. It will be of interest to identify the molecular mechanism underlying this co-operative response.

In contrast to the murine IVM studies, a recent study by Summers et al. (25), using autologous radiolabeled cells showed that unprimed human neutrophils have a transit time of 14.2 s through the lung and < 5% were retained at first pass, suggesting that a marginated population does not exist in healthy human lungs (25). However, under intravascular stimulation with LPS (2ng/kg), human volunteers showed blood neutropenia and a concomitant increase of CD11b expression on circulating neutrophils (4). Moreover, 97% of *ex vivo* primed neutrophils were retained in the lung on first pass (25). Therefore, there is no doubt that systemic challenge with LPS in humans would cause a rapid and dramatic activation of neutrophils that would then form clusters in the lung. Whether systemic *E. coli* would also be captured and retained by endothelial cells in the human lung microvascular in the absence of marginated neutrophils is currently unknown.

In the context of the spleen, tissue resident neutrophils cooperate with several resident cells to ensure confinement of pathogens. During systemic *E. coli* challenge, we show that macrophages-depletion abolishes entrapment of *E. coli* in the MZ while neutrophils in the red pulp are still able to capture *E. coli*, they fail to relocate to the MZ and cluster. Thus, in contrast to the lung, splenic resident macrophages are critically involved in orchestrating a neutrophil response to *E. coli*. In a model of listeria infection, bacteria were rapidly captured by DCs present in the splenic red pulp and neutrophils were observed to converge and aggregate around the pathogens to block spread of bacteria (26). The molecular mechanism involved in neutrophil clustering is pathogen- and tissue-specific (19, 27).

The aim of our study was to examine the dynamics of the neutrophil response to *E.coli* in multiple tissues and investigate the molecular mechanisms. To do this we used heat-killed *E. coli*. However, using live *E. coli* K-12 strain, Smith et al. (28) showed that as early as 20 min *E. coli* were found in the spleen confirming similar dynamics with heat-inactivated *E. coli* used in this study.

Stromal cells in the spleen express ICAM-1 constitutively and are the primary source of CXCL12 (21). Interestingly we show here that following *E. coli* challenge there is a dramatic reduction in both ICAM-1 expression and levels of CXCL12. The functional significance of these observations is unclear. Indeed previous work (29, 30) has shown that the CXCR4 antagonist, Plerixafor, does not stimulate the release of neutrophils from the spleen (29), consistent with the fact that in these studies the number of splenic resident neutrophils remained constant despite the down regulation of CXCL12, suggesting that the CXCL12/CXCR4 pathway is not critical for the retention of splenic neutrophils. A partial role for CD29 in the retention of splenic neutrophils has been shown by Deniset et al.- We have identified a critical role for CD29 in the re-location of neutrophils from the red pulp to the MZ.

Taken together, our data show that the same subset of neutrophils, Ly6G^high^, depending on their location, participate to pathogen recognition and capture in a tissue-dependent manner. By comparing gene, mRNA and protein expression of neutrophils in the BM, circulation, lung and spleen, it has been shown that 4 different clusters exist (31). The study by Ballesteros et al., corroborate the difference in dynamics that we observe in this study among circulating and tissue retained neutrophils. It is still an open question as to whether neutrophils are created equally or subsets of neutrophils exit the BM with pre-fixed functions and place of action (32). Studies in chronic inflammation have revealed the existence of subsets of neutrophils that are not present under homeostasis pointing towards the idea that tissues can prime or modify neutrophil phenotypes (33). We speculate that the different behaviors of splenic and circulating Ly6G^high^ and Ly6G^int^ neutrophils could be due to organ “education” or priming the retained neutrophils for a quicker and more efficient response to pathogens. Moreover, mature but not activated circulating or splenic Ly6G^high^ CD11b^+^ neutrophils show no ability to recognize and capture *E. coli* particles despite their higher level of maturity when compared with Ly6G^int^ neutrophils. Ly6G^high^ CD11b^+^ neutrophils could potentially constitute another subset of splenic neutrophils less prone to getting activated in the time frame we studied.

There are multiple studies in the literature showing neutrophil subsets and their specific dynamics and functions in different organs (34). Specifically, in the spleen, Deniset et al. (5) showed that both subsets of neutrophils, such as Ly6G^high^ and Ly6G^int^ cooperate in the clearance of gram+ *S. pneumoniae*. In our study we showed that Ly6G^high^ were the major contributors against a gram- *E. coli* challenge by up-regulating CD11b and migrating from the red pulp to the MZ to cluster. We also show that to some extent splenic Ly6G^int^ neutrophils cooperate in the capture of *E. coli* particles, while circulating Ly6G^int^ had a negligible contribution. However, splenic Ly6G^int^ failed to form clusters in the absence of splenic Ly6G^high^ neutrophils, showing their limited capacity, in response to this pathogen.

It is important to note for this study we have used female mice. Sexual dimorphism has been shown with more frequent and severe infectious diseases in males and a stronger immune response in females (35). Moreover, Kay et al. (36), showed that male mice have a far greater storage of splenic neutrophils despite similar spleen size. Dimorphism has also been described for human disease and hormones could possibly explain some of the differences (37). Therefore, understanding the activation, retention and trafficking of neutrophils in these different tissues in response to systemic E.*coli* infection in males and females could be fundamental in the designing of tailored drug therapies.

In conclusion, by examining the kinetics of the response to *E. coli*, our data suggest that tissue marginated Ly6G^high^ neutrophils are pioneer immune cells responsible for the initial capture of systemic pathogens within seconds while circulating Ly6G^high^ neutrophils only subsequently contribute to cluster enlargement and confinement of *E. coli* in the lung. Splenic Ly6G^int^ neutrophils only marginally participate in the capture of *E. coli*. Moreover, ICAM-1 is down regulated during challenge allowing splenic neutrophils to relocate from the red pulp to the MZ in a CD29-dependent migration.

## Data Availability Statement

The raw data supporting the conclusions of this article will be made available by the authors, without undue reservation to any qualified researcher by the corresponding author.

## Ethics Statement

All animal procedures and care conformed strictly to the UK Home Office. Guidelines under the Animals (Scientific Procedures) Act 1986, and the protocols were approved by the Home Office of Great Britain.

## Author Contributions

GJ performed flow cytometry analysis and PCLS/PCSS staining and analysis. JS, JV, JM and KF performed L-IVM, YH preformed 3D *ex vivo* culture, CH established the PCLS, JH helped to establish FACS analysis, and LC established L-IVM. KF established and performed S-IVM, analyzed IVMs data and generated videos. KF and SR conceived the project. SR and KF wrote the manuscript, which was edited by all authors. All authors contributed to the article and approved the submitted version.

## Funding

This work was funded by Wellcome Trust (201356/Z/16/Z). JS is funded by an Imperial College London President’s PhD Scholarship. JV is funded by Emil Aaltonen Foundation, Sigrid Juselius Foundation and Jane and Aatos Erkko Foundation. JM is supported by the NHLI foundation PhD studentship, Imperial College London. CH is supported from the Leverhulme Trust (RPG-2015-226). LC is supported by core funding from Cancer Research UK (A23983 and A17196). KF is supported by funding from the Wellcome Trust (201356/Z/16/Z).

## Conflict of Interest

The authors declare that the research was conducted in the absence of any commercial or financial relationships that could be construed as a potential conflict of interest.

## References

[1] Casanova-Acebes M Nicolas-Avila JA Li JL Garcia-Silva S Balachander A Rubio-Ponce A. Neutrophils instruct homeostatic and pathological states in naive tissues. J Exp Med (2018) 215(11):2778–95. 10.1084/jem.20181468 PMC6219739 30282719

[2] Kubes P. The enigmatic neutrophil: what we do not know. Cell Tissue Res (2018) 371(3):399–406. 10.1007/s00441-018-2790-5 29404726

[3] Looney MR Thornton EE Sen D Lamm WJ Glenny RW Krummel MF. Stabilized imaging of immune surveillance in the mouse lung. Nat Methods (2011) 8(1):91–6. 10.1038/nmeth.1543 PMC3076005 21151136

[4] Yipp BG Kim JH Lima R Zbytnuik LD Petri B Swanlund N. The Lung is a Host Defense Niche for Immediate Neutrophil-Mediated Vascular Protection. Sci Immunol (2017) 2(10). 10.1126/sciimmunol.aam8929 PMC5472445 28626833

[5] Deniset JF Surewaard BG Lee WY Kubes P. Splenic Ly6G(high) mature and Ly6G(int) immature neutrophils contribute to eradication of S. pneumoniae. J Exp Med (2017) 214(5):1333–50. 10.1084/jem.20161621 PMC5413339 28424248

[6] Altamura M Caradonna L Amati L Pellegrino NM Urgesi G Miniello S. Splenectomy and sepsis: the role of the spleen in the immune-mediated bacterial clearance. Immunopharmacol Immunotoxicol (2001) 23(2):153–61. 10.1081/IPH-100103856 11417844

[7] Sheikha AK Salih ZT Kasnazan KH Khoshnaw MK Al-Maliki T Al-Azraqi TA. Prevention of overwhelming postsplenectomy infection in thalassemia patients by partial rather than total splenectomy. Can J Surg (2007) 50(5):382–6. PMC2386178 18031639

[8] Almdahl SM Bogwald J Hoffman J Sjunneskog C Seljelid R. The effect of splenectomy on Escherichia coli sepsis and its treatment with semisoluble aminated glucan. Scand J Gastroenterol (1987) 22(3):261–7. 10.3109/00365528709078589 3296131

[9] Aichele P Zinke J Grode L Schwendener RA Kaufmann SH Seiler P. Macrophages of the splenic marginal zone are essential for trapping of blood-borne particulate antigen but dispensable for induction of specific T cell responses. J Immunol (2003) 171(3):1148–55. 10.4049/jimmunol.171.3.1148 12874200

[10] Headley MB Bins A Nip A Roberts EW Looney MR Gerard A. Visualization of immediate immune responses to pioneer metastatic cells in the lung. Nature (2016) 531(7595):513–7. 10.1038/nature16985 PMC4892380 26982733

[11] Duarte D Hawkins ED Akinduro O Ang H De Filippo K Kong IY. Inhibition of Endosteal Vascular Niche Remodeling Rescues Hematopoietic Stem Cell Loss in AML. Cell Stem Cell (2018) 22(1):64–77 e6. 10.1016/j.stem.2017.11.006 29276143 PMC5766835

[12] Akram KM Yates LL Mongey R Rothery S Gaboriau DCA Sanderson J. Live imaging of alveologenesis in precision-cut lung slices reveals dynamic epithelial cell behaviour. Nat Commun (2019) 10(1):1178. 10.1038/s41467-019-09067-3 30862802 PMC6414680

[13] Singh NR Johnson A Peters AM Babar J Chilvers ER Summers C. Acute lung injury results from failure of neutrophil de-priming: a new hypothesis. Eur J Clin Invest (2012) 42(12):1342–9. 10.1111/j.1365-2362.2012.02720.x 22984929

[14] Morris DH Bullock FD. The Importance of the Spleen in Resistance to Infection. Ann Surg (1919) 70(5):513–21. 10.1097/00000658-191911000-00001 PMC1410445 17864185

[15] Mebius RE Kraal G. Structure and function of the spleen. Nat Rev Immunol (2005) 5(8):606–16. 10.1038/nri1669 16056254

[16] Borges da Silva H Fonseca R Pereira RM Cassado Ados A Alvarez JM D’Imperio Lima MR. Splenic Macrophage Subsets and Their Function during Blood-Borne Infections. Front Immunol (2015) 6:480. 10.3389/fimmu.2015.00480 26441984 PMC4585205

[17] Perez OA Yeung ST Vera-Licona P Romagnoli PA Samji T Ural BB. CD169(+) macrophages orchestrate innate immune responses by regulating bacterial localization in the spleen. Sci Immunol (2017) 2(16). 10.1126/sciimmunol.aah5520 PMC5969998 28986418

[18] Jang JE Hod EA Spitalnik SL Frenette PS. CXCL1 and its receptor, CXCR2, mediate murine sickle cell vaso-occlusion during hemolytic transfusion reactions. J Clin Invest (2011) 121(4):1397–401. 10.1172/JCI45336 PMC3069787 21383500

[19] Lammermann T Afonso PV Angermann BR Wang JM Kastenmuller W Parent CA. Neutrophil swarms require LTB4 and integrins at sites of cell death in vivo. Nature (2013) 498(7454):371–5. 10.1038/nature12175 PMC3879961 23708969

[20] Simon SI Hu Y Vestweber D Smith CW. Neutrophil tethering on E-selectin activates beta 2 integrin binding to ICAM-1 through a mitogen-activated protein kinase signal transduction pathway. J Immunol (2000) 164(8):4348–58. 10.4049/jimmunol.164.8.4348 10754335

[21] Mueller SN Germain RN. Stromal cell contributions to the homeostasis and functionality of the immune system. Nat Rev Immunol (2009) 9(9):618–29. 10.1038/nri2588 PMC2785037 19644499

[22] Kang CI Kim SH Park WB Lee KD Kim HB Kim EC. Bloodstream infections due to extended-spectrum beta-lactamase-producing Escherichia coli and Klebsiella pneumoniae: risk factors for mortality and treatment outcome, with special emphasis on antimicrobial therapy. Antimicrob Agents Chemother (2004) 48(12):4574–81. 10.1128/AAC.48.12.4574-4581.2004 PMC529180 15561828

[23] Lien DC Wagner WW Jr Capen RL Haslett C Hanson WL Hofmeister SE. Physiological neutrophil sequestration in the lung: visual evidence for localization in capillaries. J Appl Physiol (1987) 62(3):1236–43. 10.1152/jappl.1987.62.3.1236 3106311

[24] Ekpenyong AE Toepfner N Fiddler C Herbig M Li W Cojoc G. Mechanical deformation induces depolarization of neutrophils. Sci Adv (2017) 3(6):e1602536. 10.1126/sciadv.1602536 28630905 PMC5470826

[25] Summers C Singh NR White JF Mackenzie IM Johnston A Solanki C. Pulmonary retention of primed neutrophils: a novel protective host response, which is impaired in the acute respiratory distress syndrome. Thorax (2014) 69(7):623–9. 10.1136/thoraxjnl-2013-204742 PMC4055272 24706039

[26] Waite JC Leiner I Lauer P Rae CS Barbet G Zheng H. Dynamic imaging of the effector immune response to listeria infection in vivo. PloS Pathog (2011) 7(3):e1001326. 10.1371/journal.ppat.1001326 21455492 PMC3063765

[27] Kienle K Lammermann T. Neutrophil swarming: an essential process of the neutrophil tissue response. Immunol Rev (2016) 273(1):76–93. 10.1111/imr.12458 27558329

[28] Smith SN Hagan EC Lane MC Mobley HL. Dissemination and systemic colonization of uropathogenic Escherichia coli in a murine model of bacteremia. mBio (2010) 1(5). 10.1128/mBio.00262-10 PMC2993011 21116344

[29] Pillay J Tregay N Juzenaite G Carlin LM Pirillo C Gaboriau DCA. Effect of the CXCR4 antagonist plerixafor on endogenous neutrophil dynamics in the bone marrow, lung and spleen. J Leukoc Biol (2020) 107(6):1175–85. 10.1002/JLB.1MA0420-571RR 32374077

[30] Liu Q Li Z Gao JL Wan W Ganesan S McDermott DH. CXCR4 antagonist AMD3100 redistributes leukocytes from primary immune organs to secondary immune organs, lung, and blood in mice. Eur J Immunol (2015) 45(6):1855–67. 10.1002/eji.201445245 PMC4461468 25801950

[31] Ballesteros I Rubio-Ponce A Genua M Lusito E Kwok I Fernandez-Calvo G. Co-option of Neutrophil Fates by Tissue Environments. Cell (2020) 183(5):1282–97. 10.1016/j.cell.2020.10.003 33098771

[32] Ley K Hoffman HM Kubes P Cassatella MA Zychlinsky A Hedrick CC. Neutrophils: New insights and open questions. Sci Immunol (2018) 3(30):183(5):1282–97. 10.1126/sciimmunol.aat4579 30530726

[33] Tsuda Y Takahashi H Kobayashi M Hanafusa T Herndon DN Suzuki F. Three different neutrophil subsets exhibited in mice with different susceptibilities to infection by methicillin-resistant Staphylococcus aureus. Immunity (2004) 21(2):215–26. 10.1016/j.immuni.2004.07.006 15308102

[34] De Filippo K Rankin SM. The Secretive Life of Neutrophils Revealed by Intravital Microscopy. Front Cell Dev Biol (2020) 8:603230. 10.3389/fcell.2020.603230 33240898 PMC7683517

[35] Gal-Oz ST Maier B Yoshida H Seddu K Elbaz N Czysz C. ImmGen report: sexual dimorphism in the immune system transcriptome. Nat Commun (2019) 10(1):4295. 10.1038/s41467-019-12348-6 31541153 PMC6754408

[36] Kay E Gomez-Garcia L Woodfin A Scotland RS Whiteford JR. Sexual dimorphisms in leukocyte trafficking in a mouse peritonitis model. J Leukoc Biol (2015) 98(5):805–17. 10.1189/jlb.3A1214-601RR 26138922

[37] Marriott I Huet-Hudson YM. Sexual dimorphism in innate immune responses to infectious organisms. Immunol Res (2006) 34(3):177–92. 10.1385/IR:34:3:177 16891670

