# Supplementary figures and images for: Lung Marginated and Splenic Murine Resident Neutrophils Constitute Pioneers in Tissue-Defense During Systemic E. coli Challenge
Source: Front Immunol. 2021 Apr 19;12:597595. doi: 10.3389/fimmu.2021.597595 (PMC8089477; doi:10.3389/fimmu.2021.597595)

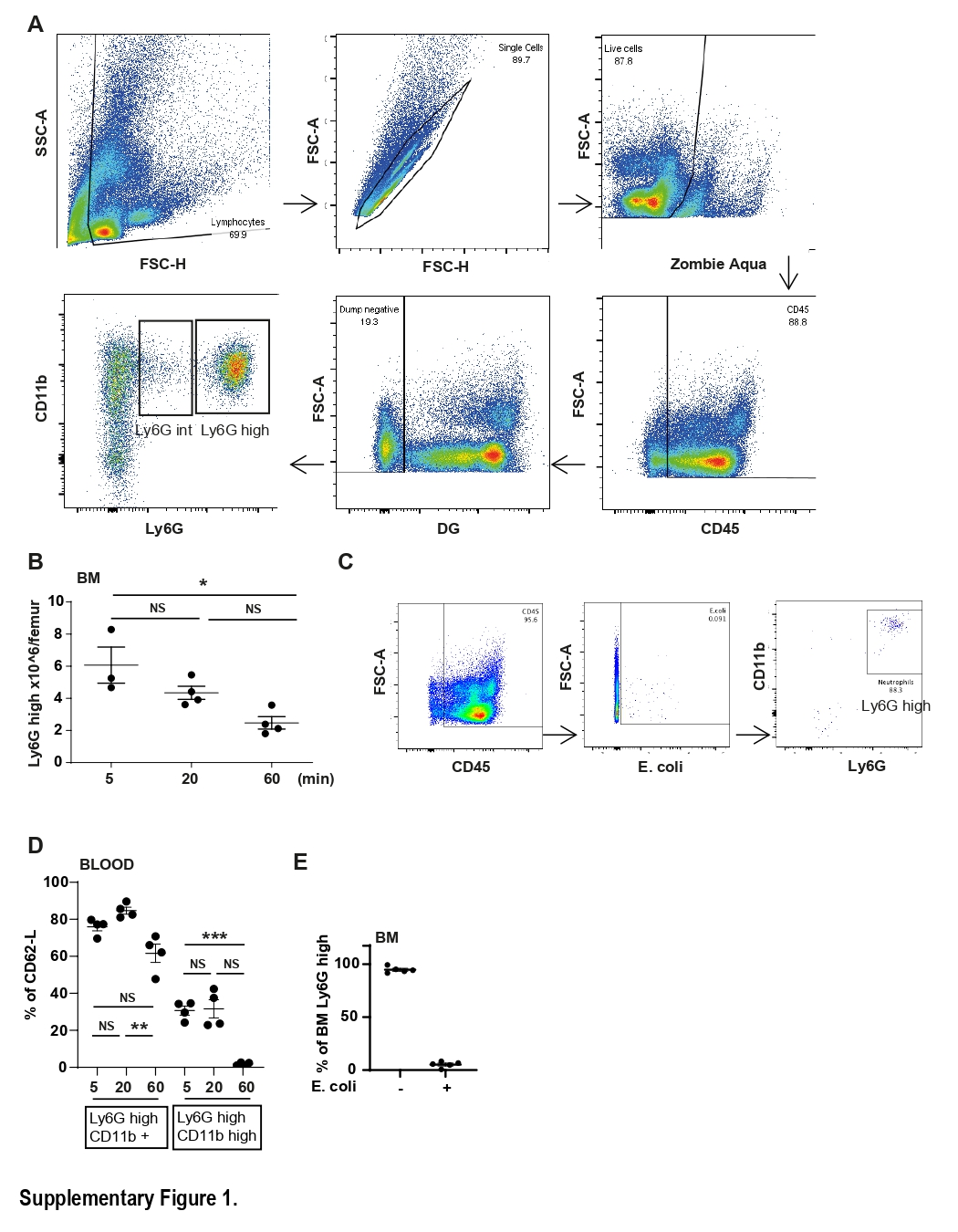

Supplement: Supplementary file 1 [file Image_1.jpeg]

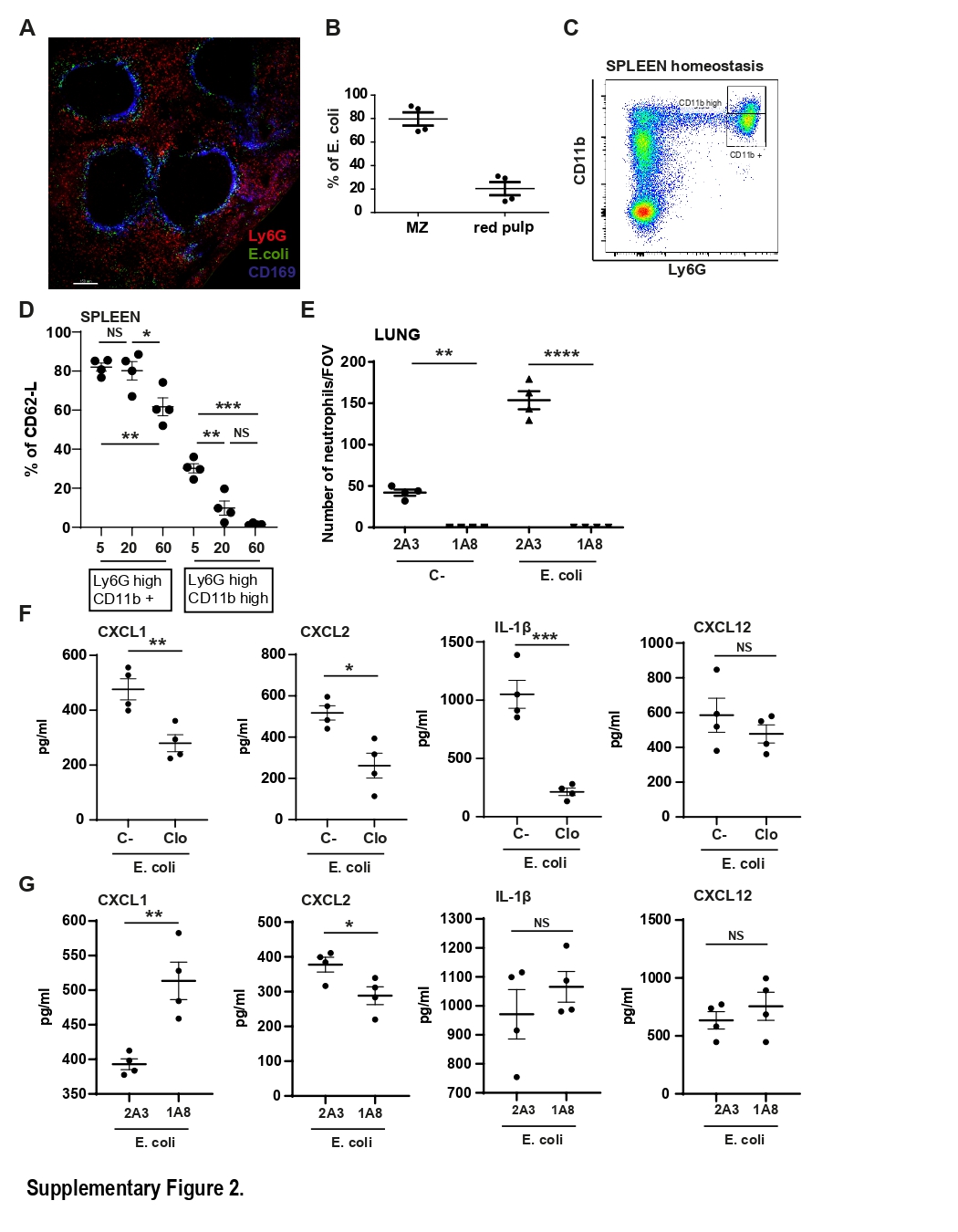

Supplement: Supplementary file 2 [file Image_2.jpeg]
